# (*Z*)-Ethyl 3-(2,4,6-trimethyl­anilino)but-2-enoate

**DOI:** 10.1107/S160053680903949X

**Published:** 2009-10-17

**Authors:** Manuel Amézquita-Valencia, Simón Hernández-Ortega, G. Alejandra Suárez-Ortiz, Rubén Alfredo Toscano, Armando Cabrera

**Affiliations:** a Instituto de Química, Universidad Nacional Autónoma de México, Circuito Exterior, Ciudad Universitaria, México 04510, Mexico

## Abstract

The title compound, C_15_H_21_NO_2_, was obtained by the reaction of acetoacetate with 2,4,6-trimethyl­aniline using Mexican bentonitic clay as a catalyst. It crystallizes in the enamine form. The β-enamino ester residue is almost perpendicular to the aromatic ring [dihedral angle = 88.10 (6)°]. The mol­ecular conformation is stabilized by a strong intra­molecular N—H⋯O hydrogen bond. In addition, the N—H group forms a weak inter­molecular N—H⋯O hydrogen bond linking the mol­ecules into centrosymmetric dimers.

## Related literature

For enamino esters as inter­mediates in the synthesis of natural products, see: Marchand *et al.* (1994 ▶). β-Enamino esters are useful in synthesis of pharmaceuticals and bioactive heterocycles (Spivey *et al.*, 2003 ▶) and as precursors for the preparation of anti­bacterial, anti­convulsant (Michael *et al.*, 2001 ▶), anti-inflamatory and anti­tumour agents. For the functionalization of these compounds by the introduction of different substituents on the nitro­gen, α-carbon and β-carbonylic carbon atoms, see: Braibante *et al.* (2002 ▶).
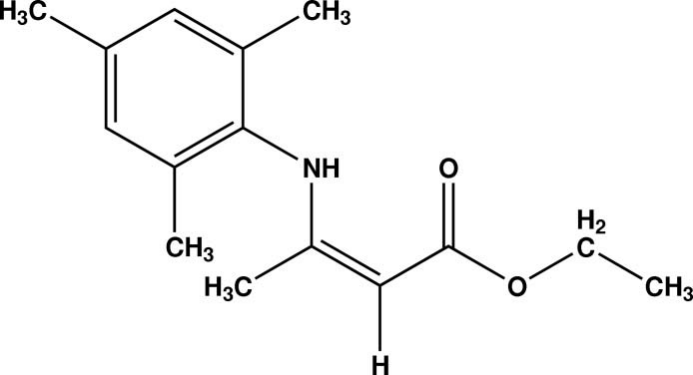


## Experimental

### 

#### Crystal data

C_15_H_21_NO_2_
*M* *_r_* = 247.33
Monoclinic, 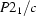

*a* = 8.5647 (8) Å
*b* = 20.6131 (19) Å
*c* = 8.2404 (8) Å
β = 93.976 (2)°
*V* = 1451.3 (2) Å^3^
*Z* = 4
Mo *K*α radiation
μ = 0.07 mm^−1^
*T* = 298 K
0.48 × 0.37 × 0.15 mm

#### Data collection

Bruker SMART APEX CCD diffractometer
Absorption correction: multi-scan (*SADABS*; Bruker, 1999 ▶) *T*_min_ = 0.970, *T*_max_ = 0.989
11717 measured reflections
2634 independent reflections
2160 reflections with *I* > 2σ(*I*)
*R*_int_ = 0.028

#### Refinement

*R*[*F* ^2^ > 2σ(*F* ^2^)] = 0.049
*wR*(*F* ^2^) = 0.145
*S* = 1.05
2634 reflections
166 parameters
H atoms treated by a mixture of independent and constrained refinement
Δρ_max_ = 0.19 e Å^−3^
Δρ_min_ = −0.21 e Å^−3^

### 

Data collection: *SMART* (Bruker, 1999 ▶); cell refinement: *SAINT* (Bruker, 1999 ▶); data reduction: *SAINT*; program(s) used to solve structure: *SHELXTL* (Sheldrick, 2008 ▶); program(s) used to refine structure: *SHELXTL*; molecular graphics: *SHELXTL*; software used to prepare material for publication: *SHELXTL*.

## Supplementary Material

Crystal structure: contains datablocks I, global. DOI: 10.1107/S160053680903949X/bt5072sup1.cif

Structure factors: contains datablocks I. DOI: 10.1107/S160053680903949X/bt5072Isup2.hkl

Additional supplementary materials:  crystallographic information; 3D view; checkCIF report

## Figures and Tables

**Table 1:** Hydrogen-bond geometry (Å, °)

*D*—H⋯*A*	*D*—H	H⋯*A*	*D*⋯*A*	*D*—H⋯*A*
N1—H1⋯O1	0.82 (2)	2.08 (2)	2.7516 (18)	138.7 (17)
N1—H1⋯O1^i^	0.82 (2)	2.60 (2)	3.2201 (18)	133.1 (16)

Symmetry code: (i) 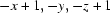.

**Fig. 1.**
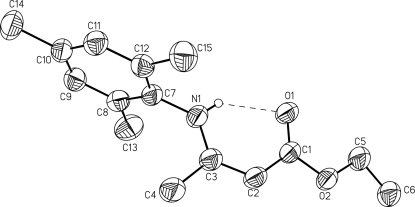
The Molecular structure with the atom numbering scheme. Displacement ellipsoids are drawn at the 40% probability level. H atoms bonded to C omitted. The intramolecular hydrogen bond is shown as a dashed line.

## supplementary crystallographic information

### Comment

The enamino esters are gaining increased interest, which are known as important intermediates for the synthesis of natural products (Marchand *et  al*, 1994). The β-enamino esters are useful in synthesis of pharmaceuticals and bioactive heterocycles (Spivey *et al.*, 2003) and as precursors for the preparation of antibacterial, anticonvulsant (Michael *et al.*, 2001), anti-inflamatory and antitumour agents. The functionalization of these compounds by the introduction of different substituents on the nitrogen atom, the α-carbon and β-carbonylic carbon atoms has been studied (Braibante *et al.*, 2002).

The molecular structure and the atomic numbering scheme is shown in Fig. 1.     The trimethylyphenyl substituent is almost perpendicular to the β-enaminoester function forming a dihedral angle of 88.10 (6)°.

### Experimental

A mixture of ethyl acetoacetate (5 mmol), 2,4,6-trimethylaniline (5 mmol) were dispersed on Actisil-FF (1 g, Mexican Bentonitic Clay) and the mixture was stirred at r.t   overnight. The product was extracted by washing the clay with CH_2_Cl_2_ (3x10mL), dried (Na_2_SO_4_), filtred and the solvent was removed *in vacuo*. The crude product was purified by column chromatography and recrystallized from hexane. Yield: 92%, *M.p. 65.4°C*

### Refinement

H atom on amine group was found in Fourier map and its coordinates were refined with *U*_iso_(H) = 1.2 U_eq_(N). H atoms bonded to C atoms were placed in geometrically idealized positions [C-H = 0.97 Å (for CH_2_) and 0.96 Å (for CH_3_)] and refined using a riding model with *U*_iso_(H) = 1.2 U_eq_(C) or 1.5 U_eq_C(methyl).

### Crystal data

**Table:**

C_15_H_21_NO_2_	*F*(000) = 536
*M**_r_* = 247.33	*D*_x_ = 1.132 Mg m^−^^3^
Monoclinic, *P*2_1_/*c*	Melting point: 338.2 K
Hall symbol: -P 2ybc	Mo *K*α radiation, λ = 0.71073 Å
*a* = 8.5647 (8) Å	Cell parameters from 5426 reflections
*b* = 20.6131 (19) Å	θ = 2.6–25.3°
*c* = 8.2404 (8) Å	µ = 0.07 mm^−^^1^
β = 93.976 (2)°	*T* = 298 K
*V* = 1451.3 (2) Å^3^	Plates, colorless
*Z* = 4	0.48 × 0.37 × 0.15 mm

### Data collection

**Table:**

Bruker SMART APEX CCD diffractometer	2634 independent reflections
Radiation source: fine-focus sealed tube	2160 reflections with *I* > 2σ(*I*)
graphite	*R*_int_ = 0.028
Detector resolution: 0.661 pixels mm^-1^	θ_max_ = 25.4°, θ_min_ = 2.0°
ω scans	*h* = −10→10
Absorption correction: multi-scan (*SADABS*; Bruker, 1999)	*k* = −24→24
*T*_min_ = 0.970, *T*_max_ = 0.989	*l* = −9→9
11717 measured reflections	

### Refinement

**Table:**

Refinement on *F*^2^	Primary atom site location: structure-invariant direct methods
Least-squares matrix: full	Secondary atom site location: difference Fourier map
*R*[*F*^2^ > 2σ(*F*^2^)] = 0.049	Hydrogen site location: inferred from neighbouring sites
*wR*(*F*^2^) = 0.145	H atoms treated by a mixture of independent and constrained refinement
*S* = 1.05	*w* = 1/[σ^2^(*F*_o_^2^) + (0.0824*P*)^2^ + 0.188*P*] where *P* = (*F*_o_^2^ + 2*F*_c_^2^)/3
2634 reflections	(Δ/σ)_max_ < 0.001
166 parameters	Δρ_max_ = 0.19 e Å^−^^3^
0 restraints	Δρ_min_ = −0.21 e Å^−^^3^

### Special details

**Table:**

Geometry. All e.s.d.'s (except the e.s.d. in the dihedral angle between two l.s. planes) are estimated using the full covariance matrix. The cell e.s.d.'s are taken into account individually in the estimation of e.s.d.'s in distances, angles and torsion angles; correlations between e.s.d.'s in cell parameters are only used when they are defined by crystal symmetry. An approximate (isotropic) treatment of cell e.s.d.'s is used for estimating e.s.d.'s involving l.s. planes.
Refinement. Refinement of *F*^2^ against ALL reflections. The weighted *R*-factor *wR* and goodness of fit *S* are based on *F*^2^, conventional *R*-factors *R* are based on *F*, with *F* set to zero for negative *F*^2^. The threshold expression of *F*^2^ > σ(*F*^2^) is used only for calculating *R*-factors(gt) *etc*. and is not relevant to the choice of reflections for refinement. *R*-factors based on *F*^2^ are statistically about twice as large as those based on *F*, and *R*- factors based on ALL data will be even larger.

### Fractional atomic coordinates and isotropic or equivalent isotropic displacement parameters (Å^2^)

**Table:**

	*x*	*y*	*z*	*U*_iso_*/*U*_eq_	
O1	0.34094 (14)	0.02629 (6)	0.57044 (15)	0.0678 (4)	
O2	0.12297 (13)	0.06508 (5)	0.67115 (14)	0.0634 (3)	
N1	0.48468 (17)	0.11091 (7)	0.36990 (19)	0.0608 (4)	
H1	0.475 (2)	0.0741 (10)	0.407 (2)	0.073*	
C1	0.25234 (18)	0.07166 (8)	0.58833 (18)	0.0531 (4)	
C2	0.26799 (18)	0.13592 (8)	0.5250 (2)	0.0572 (4)	
H2	0.1988	0.1676	0.5560	0.069*	
C3	0.37828 (18)	0.15303 (8)	0.4223 (2)	0.0559 (4)	
C4	0.3850 (2)	0.22125 (9)	0.3585 (3)	0.0767 (6)	
H4A	0.4923	0.2344	0.3555	0.115*	
H4B	0.3321	0.2499	0.4285	0.115*	
H4C	0.3349	0.2230	0.2507	0.115*	
C5	0.0901 (2)	0.00058 (9)	0.7287 (3)	0.0746 (5)	
H5A	0.1642	−0.0108	0.8185	0.089*	
H5B	0.0998	−0.0307	0.6421	0.089*	
C6	−0.0711 (2)	−0.00040 (10)	0.7828 (2)	0.0733 (5)	
H6A	−0.0964	−0.0435	0.8164	0.110*	
H6B	−0.1433	0.0128	0.6946	0.110*	
H6C	−0.0782	0.0289	0.8725	0.110*	
C7	0.60799 (18)	0.12825 (7)	0.27088 (19)	0.0520 (4)	
C8	0.58802 (19)	0.12037 (8)	0.1023 (2)	0.0568 (4)	
C9	0.7112 (2)	0.13715 (8)	0.0097 (2)	0.0603 (4)	
H9	0.6984	0.1328	−0.1027	0.072*	
C10	0.85176 (19)	0.16000 (8)	0.0787 (2)	0.0568 (4)	
C11	0.86788 (19)	0.16666 (8)	0.2457 (2)	0.0577 (4)	
H11	0.9623	0.1819	0.2938	0.069*	
C12	0.74836 (19)	0.15143 (7)	0.34424 (19)	0.0549 (4)	
C13	0.4379 (2)	0.09401 (11)	0.0225 (3)	0.0857 (6)	
H13A	0.4511	0.0857	−0.0903	0.129*	
H13B	0.3556	0.1251	0.0319	0.129*	
H13C	0.4112	0.0544	0.0752	0.129*	
C14	0.9846 (2)	0.17663 (10)	−0.0250 (3)	0.0794 (6)	
H14A	1.0610	0.2021	0.0375	0.119*	
H14B	0.9449	0.2010	−0.1182	0.119*	
H14C	1.0322	0.1374	−0.0603	0.119*	
C15	0.7719 (3)	0.15816 (11)	0.5262 (2)	0.0779 (6)	
H15A	0.8812	0.1640	0.5567	0.117*	
H15B	0.7350	0.1197	0.5771	0.117*	
H15C	0.7146	0.1951	0.5608	0.117*	

### Atomic displacement parameters (Å^2^)

**Table:**

	*U*^11^	*U*^22^	*U*^33^	*U*^12^	*U*^13^	*U*^23^
O1	0.0634 (7)	0.0594 (7)	0.0838 (8)	0.0124 (6)	0.0279 (6)	0.0107 (6)
O2	0.0624 (7)	0.0567 (7)	0.0746 (7)	0.0074 (5)	0.0297 (6)	0.0078 (5)
N1	0.0599 (8)	0.0490 (8)	0.0766 (9)	0.0057 (6)	0.0279 (7)	0.0094 (6)
C1	0.0497 (8)	0.0573 (9)	0.0535 (8)	0.0036 (7)	0.0120 (7)	−0.0022 (6)
C2	0.0530 (9)	0.0534 (9)	0.0672 (10)	0.0071 (7)	0.0176 (7)	−0.0019 (7)
C3	0.0543 (9)	0.0506 (8)	0.0639 (9)	0.0035 (7)	0.0119 (7)	0.0002 (7)
C4	0.0776 (12)	0.0550 (10)	0.1014 (14)	0.0119 (9)	0.0333 (11)	0.0115 (9)
C5	0.0786 (12)	0.0627 (11)	0.0861 (12)	0.0072 (9)	0.0319 (10)	0.0177 (9)
C6	0.0698 (12)	0.0707 (11)	0.0814 (12)	−0.0063 (9)	0.0192 (9)	0.0079 (9)
C7	0.0514 (9)	0.0451 (8)	0.0610 (9)	0.0051 (6)	0.0156 (7)	0.0047 (6)
C8	0.0527 (9)	0.0570 (9)	0.0610 (9)	0.0046 (7)	0.0056 (7)	−0.0013 (7)
C9	0.0667 (10)	0.0639 (10)	0.0510 (8)	0.0071 (8)	0.0102 (7)	0.0015 (7)
C10	0.0594 (10)	0.0492 (8)	0.0636 (9)	0.0036 (7)	0.0186 (7)	0.0058 (7)
C11	0.0534 (9)	0.0518 (9)	0.0683 (10)	−0.0035 (7)	0.0079 (7)	0.0008 (7)
C12	0.0610 (10)	0.0492 (8)	0.0552 (9)	0.0056 (7)	0.0081 (7)	0.0026 (6)
C13	0.0639 (12)	0.1009 (16)	0.0914 (14)	−0.0044 (10)	−0.0012 (10)	−0.0153 (12)
C14	0.0781 (13)	0.0738 (12)	0.0907 (14)	−0.0036 (10)	0.0375 (11)	0.0090 (10)
C15	0.0872 (14)	0.0882 (14)	0.0583 (10)	0.0039 (11)	0.0061 (9)	−0.0009 (9)

### Geometric parameters (Å, °)

**Table:**

O1—C1	1.2196 (18)	C7—C8	1.397 (2)
O2—C1	1.3479 (18)	C8—C9	1.388 (2)
O2—C5	1.446 (2)	C8—C13	1.505 (3)
N1—C3	1.351 (2)	C9—C10	1.378 (2)
N1—C7	1.4243 (19)	C9—H9	0.9300
N1—H1	0.82 (2)	C10—C11	1.380 (2)
C1—C2	1.433 (2)	C10—C14	1.509 (2)
C2—C3	1.358 (2)	C11—C12	1.386 (2)
C2—H2	0.9300	C11—H11	0.9300
C3—C4	1.504 (2)	C12—C15	1.506 (2)
C4—H4A	0.9600	C13—H13A	0.9600
C4—H4B	0.9600	C13—H13B	0.9600
C4—H4C	0.9600	C13—H13C	0.9600
C5—C6	1.481 (3)	C14—H14A	0.9600
C5—H5A	0.9700	C14—H14B	0.9600
C5—H5B	0.9700	C14—H14C	0.9600
C6—H6A	0.9600	C15—H15A	0.9600
C6—H6B	0.9600	C15—H15B	0.9600
C6—H6C	0.9600	C15—H15C	0.9600
C7—C12	1.392 (2)		
			
C1—O2—C5	116.36 (12)	C9—C8—C7	118.18 (15)
C3—N1—C7	124.42 (14)	C9—C8—C13	120.59 (16)
C3—N1—H1	112.7 (13)	C7—C8—C13	121.23 (16)
C7—N1—H1	122.8 (13)	C10—C9—C8	122.28 (15)
O1—C1—O2	121.62 (14)	C10—C9—H9	118.9
O1—C1—C2	126.15 (14)	C8—C9—H9	118.9
O2—C1—C2	112.22 (13)	C9—C10—C11	117.98 (15)
C3—C2—C1	123.61 (14)	C9—C10—C14	121.04 (16)
C3—C2—H2	118.2	C11—C10—C14	120.98 (17)
C1—C2—H2	118.2	C10—C11—C12	122.32 (15)
N1—C3—C2	123.10 (15)	C10—C11—H11	118.8
N1—C3—C4	116.49 (15)	C12—C11—H11	118.8
C2—C3—C4	120.40 (14)	C11—C12—C7	118.29 (15)
C3—C4—H4A	109.5	C11—C12—C15	120.61 (16)
C3—C4—H4B	109.5	C7—C12—C15	121.08 (15)
H4A—C4—H4B	109.5	C8—C13—H13A	109.5
C3—C4—H4C	109.5	C8—C13—H13B	109.5
H4A—C4—H4C	109.5	H13A—C13—H13B	109.5
H4B—C4—H4C	109.5	C8—C13—H13C	109.5
O2—C5—C6	108.55 (15)	H13A—C13—H13C	109.5
O2—C5—H5A	110.0	H13B—C13—H13C	109.5
C6—C5—H5A	110.0	C10—C14—H14A	109.5
O2—C5—H5B	110.0	C10—C14—H14B	109.5
C6—C5—H5B	110.0	H14A—C14—H14B	109.5
H5A—C5—H5B	108.4	C10—C14—H14C	109.5
C5—C6—H6A	109.5	H14A—C14—H14C	109.5
C5—C6—H6B	109.5	H14B—C14—H14C	109.5
H6A—C6—H6B	109.5	C12—C15—H15A	109.5
C5—C6—H6C	109.5	C12—C15—H15B	109.5
H6A—C6—H6C	109.5	H15A—C15—H15B	109.5
H6B—C6—H6C	109.5	C12—C15—H15C	109.5
C12—C7—C8	120.94 (14)	H15A—C15—H15C	109.5
C12—C7—N1	119.32 (14)	H15B—C15—H15C	109.5
C8—C7—N1	119.73 (14)		
			
C5—O2—C1—O1	−3.7 (2)	N1—C7—C8—C13	0.2 (2)
C5—O2—C1—C2	175.22 (15)	C7—C8—C9—C10	1.2 (2)
O1—C1—C2—C3	6.4 (3)	C13—C8—C9—C10	−178.42 (17)
O2—C1—C2—C3	−172.41 (15)	C8—C9—C10—C11	−0.6 (2)
C7—N1—C3—C2	−175.71 (16)	C8—C9—C10—C14	178.82 (16)
C7—N1—C3—C4	5.4 (3)	C9—C10—C11—C12	−0.2 (2)
C1—C2—C3—N1	−0.1 (3)	C14—C10—C11—C12	−179.62 (15)
C1—C2—C3—C4	178.75 (17)	C10—C11—C12—C7	0.4 (2)
C1—O2—C5—C6	−167.50 (15)	C10—C11—C12—C15	178.79 (16)
C3—N1—C7—C12	85.6 (2)	C8—C7—C12—C11	0.1 (2)
C3—N1—C7—C8	−95.9 (2)	N1—C7—C12—C11	178.62 (13)
C12—C7—C8—C9	−0.9 (2)	C8—C7—C12—C15	−178.23 (15)
N1—C7—C8—C9	−179.37 (13)	N1—C7—C12—C15	0.2 (2)
C12—C7—C8—C13	178.68 (16)		

### Hydrogen-bond geometry (Å, °)

**Table:**

*D*—H···*A*	*D*—H	H···*A*	*D*···*A*	*D*—H···*A*
N1—H1···O1	0.82 (2)	2.08 (2)	2.7516 (18)	138.7 (17)
N1—H1···O1^i^	0.82 (2)	2.60 (2)	3.2201 (18)	133.1 (16)

Symmetry codes: (i) −*x*+1, −*y*, −*z*+1.
